# Automatic Liver Viability Scoring with Deep Learning and Hyperspectral Imaging

**DOI:** 10.3390/diagnostics11091527

**Published:** 2021-08-24

**Authors:** Eric Felli, Mahdi Al-Taher, Toby Collins, Richard Nkusi, Emanuele Felli, Andrea Baiocchini, Veronique Lindner, Cindy Vincent, Manuel Barberio, Bernard Geny, Giuseppe Maria Ettorre, Alexandre Hostettler, Didier Mutter, Sylvain Gioux, Catherine Schuster, Jacques Marescaux, Jordi Gracia-Sancho, Michele Diana

**Affiliations:** 1 Hepatology, Department of Biomedical Research, Inselspital, University of Bern, 3008 Bern, Switzerland; jordi.gracia@idibaps.org; 2 IHU-Strasbourg, Institute of Image-Guided Surgery, 67000 Strasbourg, France; cindy.vincent@ihu-strasbourg.eu; 3 Institute of Physiology, EA3072 Mitochondria Respiration and Oxidative Stress, University of Strasbourg, 67000 Strasbourg, France; bernard.geny@chru-strasbourg.fr; 4 Research Institute against Digestive Cancer (IRCAD), 67000 Strasbourg, France; mahdi.al-taher@ircad.fr (M.A.-T.); toby.collins@ircad.fr (T.C.); ricardonkusi@gmail.com (R.N.); alexandre.hostettler@ircad.fr (A.H.); jacques.marescaux@ircad.fr (J.M.); michele.diana@ircad.fr (M.D.); 5 Department of General, Digestive, and Endocrine Surgery, University Hospital of Strasbourg, 67000 Strasbourg, France; emanuele.felli@chru-strasbourg.fr (E.F.); d.mutter@ircad.fr (D.M.); 6 Department of Pathology, San Camillo Forlanini Hospital, 00152 Rome, Italy; baiocchiniandrea@gmail.com; 7 Department of Pathology, University Hospital of Strasbourg, 67000 Strasbourg, France; veronique.lindner@chru-strasbourg.fr; 8 Department of General Surgery, Cardinale Giovanni Panico Hospital, 73039 Tricase, Italy; manuel.barberio@ircad.fr; 9 San Camillo Forlanini Hospital, Department of Transplantation and General Surgery, 00152 Rome, Italy; GmEttorre@scamilloforlanini.rm.it; 10 Photonics Instrumentation for Health, iCube Laboratory, University of Strasbourg, 67000 Strasbourg, France; sgioux@unistra.fr; 11 INSERM, Institute of Viral and Liver Disease, U1110, 67000 Strasbourg, France; catherine.schuster@unistra.fr; 12 Liver Vascular Biology, IDIBAPS Biomedical Research Institute and CIBEREHD, 08036 Barcelona, Spain

**Keywords:** liver viability, artificial intelligence, deep learning, convolutional networks, CNNs, hyperspectral imaging, hepatic artery occlusion

## Abstract

Hyperspectral imaging (HSI) is a non-invasive imaging modality already applied to evaluate hepatic oxygenation and to discriminate different models of hepatic ischemia. Nevertheless, the ability of HSI to detect and predict the reperfusion damage intraoperatively was not yet assessed. Hypoxia caused by hepatic artery occlusion (HAO) in the liver brings about dreadful vascular complications known as ischemia-reperfusion injury (IRI). Here, we show the evaluation of liver viability in an HAO model with an artificial intelligence-based analysis of HSI. We have combined the potential of HSI to extract quantitative optical tissue properties with a deep learning-based model using convolutional neural networks. The artificial intelligence (AI) score of liver viability showed a significant correlation with capillary lactate from the liver surface (r = −0.78, *p* = 0.0320) and Suzuki’s score (r = −0.96, *p* = 0.0012). CD31 immunostaining confirmed the microvascular damage accordingly with the AI score. Our results ultimately show the potential of an HSI-AI-based analysis to predict liver viability, thereby prompting for intraoperative tool development to explore its application in a clinical setting.

## 1. Introduction

During the last decade, hyperspectral imaging (HSI) has gained importance in the biomedical field [1,2,3]. HSI systems aim to build images based on the computational analysis of light-tissue interactions through the detection of relative reflectance. This allows for the quantification of organic compounds such as oxygenated and deoxygenated hemoglobin at different depths in a wide large field of view [4]. Recently applied for the quantitative analysis of liver perfusion assessment [1,5], HSI was also used for the study of arterial perfusion [6,7,8,9,10,11]. Hypoxia produced by the occlusion of arterial flow causes severe time-dependent complications. For instance, hepatic artery occlusion (HAO) is a dreadful vascular event, which can occur in different clinical scenarios such as hepatic artery thrombosis, hepatic artery ligation during liver surgery, emboli, arterial abnormalities, eclampsia, and sickle cell crisis. Oxygen deprivation is the starting event that induces parenchymal damage, which is further aggravated once blood circulation has been re-established by the ischemia-reperfusion injury mechanism (IRI) [12,13]. IRI is associated with a massive injury to hepatocytes even when diagnosed in the early phase (within 2 h) [14,15,16]. When the occlusion of the hepatic artery occurs, despite its dual vascular inflows, the liver goes under global hypoxia since the hepatic artery contributes ~50% of the oxygen supply. The portal flow alone is insufficient to prevent the occurrence of anaerobic metabolism, which can be rapidly evaluated with the analysis of capillary and systemic lactate concentration [17,18]. Consequently, warm ischemia like cold ischemia disrupts the microcirculation downregulating the sinusoidal protective phenotype [19,20,21]. As a result, liver perfusion assessment is an essential target for the diagnosis of hepatic hypoxia in both ischemic types.

Intraoperative Doppler ultrasonography (US) is the standard tool for an immediate evaluation of the HAO, followed by a daily US in combination with transaminase control until postoperative day 5 or 7. If hepatic artery thrombosis (HAT) is suspected, a contrast-enhanced CT scan or less often a magnetic resonance scan is performed to confirm the diagnosis. If US and CT or magnetic resonance do not detect HAT, angiography may be helpful [22]. However, all these techniques have some drawbacks. As a matter of fact, they are time-consuming, they require a long-term learning curve, and their interpretation may vary. Additionally, in the case of arterial revascularization, it is not possible to predict the future graft function and the potential consequences of IRI damage. Early detection or, ideally, intraoperative prediction of graft dysfunction or failure would be crucial for timely treatment.

The ongoing research to predict IRI has considered various solutions. They include indocyanine green (ICG) fluorescence imaging, near-infrared spectroscopy (NIRS), microdialysis, and carbon dioxide sensors [23,24,25,26,27,28]. However, such methods are limited by the need for the administration of an exogenous dye, and/or by some degree of invasiveness. In addition, these approaches do not provide a direct oxygenation map of the organ, which could allow for an immediate localization of liver ischemic damage.

Recently, our group has demonstrated the potential usefulness of HSI as an intraoperative tool during image-guided liver surgery [29]. In particular, HSI could intraoperatively quantify, discriminate, and visualize different types of liver ischemia, including HAO and total vascular inflow occlusion [30]. We are currently working on exploring the potential of HSI coupled with deep learning-based analysis of HS images to intraoperatively predict the ischemia-reperfusion damage. As a first step and a proof of concept, we focused on the ability of HSI to predict damage given by liver hypoxia ligating only the hepatic artery [31]. This allowed us to exclude only the oxygen variable without the more complex composition of blood supply from the portal vein.

Machine learning has recently been used for the automatic analysis of hyperspectral image data [32], mainly driven from the remote sensing community [33,34], but now extending to a range of medical applications [1] such as automatic tumor detection [35,36] and histopathological analysis [37]. However, there have not been prior studies reporting on the use of machine learning models to automatically characterize liver reperfusion damage intraoperatively in a large field of view.

Our hypothesis is that machine learning models can be trained to automatically recognize the optical properties associated with the reperfusion damage given by HAO in HSI images using supervised learning. Consequently, a predictive AI analysis can be built to provide an automatic convenient and non-invasive tool for intraoperative ischemic liver disease detection.

## 2. Materials and Methods

### 2.1. Study Design

**Figure 1:**
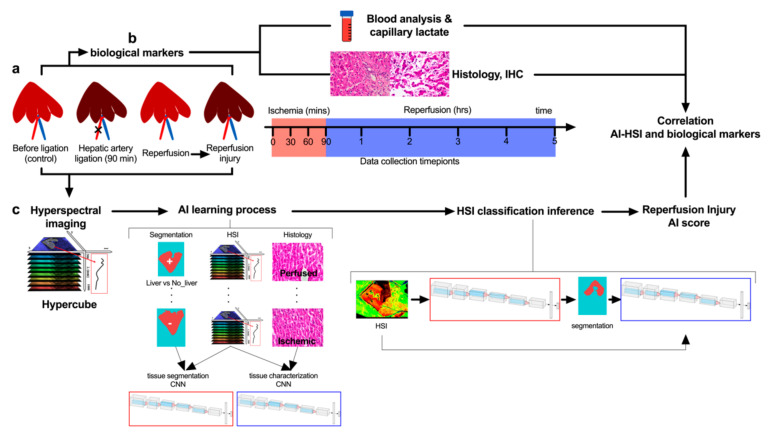
Experimental workflow. (**a**) Hepatic artery occlusion (HAO) was performed for 90 min followed by a reperfusion phase of 5 h. (**b**) During ischemia and reperfusion time, biological data and hyperspectral imaging (HSI) were sampled. (**c**) Hyperspectral imaging was acquired providing the hypercube with a wavelength range from 500 to 1000 nm. Two artificial intelligence-based convolutional neural networks (CNNs) were trained to perform a segmentation that could identify the liver surface and analyze the same surface to predict perfused and not perfused livers during the ischemia phase. Finally, the tissue classification produced by the CNNs was used to create a score of liver viability during the reperfusion phase. While StO_2_% and NIR% are calculated with the preset algorithm of the HSI (TIVITA software), the AI score is calculated over the whole spectra using the hypercube.

Sample size calculation was performed using the correlation between optical and biological data. The calculation was based on previous publications on bowel ischemia which showed a ρ correlation coefficient of −0.7 [38,39]. The required sample size in terms of paired values was 4, considering α = 0.05 with a power (1 − β) = 0.9. In the present study, 42 paired values StO_2_% and lactates values were obtained in 5 pigs in total. The AI model used a pixel by pixel (640 × 480) analysis per each of the 42 images providing a large dataset elaboration. The aim of the study was to predict liver viability through the analysis of hyperspectral images using artificial intelligence based on convolutional neural networks (CNNs) to (i) discriminate the liver from the rest of the tissues, (ii) recognize perfused from the non-perfused liver, (iii) predict the level of liver perfusion during the reperfusion phase, and (iv) predict biological data (Figure 1a–c). The control group is represented by the same treated pigs before hepatic artery ligation. The ischemic phase was held for 90 min, collecting optical and biological data every 30 min (Figure 1a,b). The following reperfusion phase was monitored for 5 h, collecting data every hour. The hypercube extracted from hyperspectral images was used to train two CNNs (Figure 1c). Finally, the generated AI score for the reperfusion phase was generated and the quantitative analysis of hyperspectral images was correlated with biological data. Histopathological evaluation and scoring were performed in a blinded fashion. Capillary lactate was sampled randomly through the liver surface.

### 2.2. Animals

The present study, which is part of the ELIOS project (Endoscopic Luminescent Imaging for Oncology Surgery), was approved by the local Ethical Committee on Animal Experimentation (ICOMETH No. 38.2016.01.085), as well as by the French Ministry of Superior Education and Research (MESR) (APAFIS#8721-2017013010316298-v2). The experimental procedure followed the general indications already published in the protocol exchange [40]. All animals used in the experiment were managed according to French laws for animal use and care, and according to the directives of the European Community Council (2010/63/EU) and ARRIVE guidelines [41]. Five adult male swine (Sus scrofa ssp. domesticus, mean weight: 32.4 ± 4.4 kg) were housed and acclimatized for 48 h in an enriched environment, respecting circadian cycles of light-darkness, with constant humidity and temperature conditions. They were fasted 24 h before surgery, with ad libitum access to water, and finally sedated (zolazepam + tiletamine 10 mg/kg IM) 30 min before the procedure to decrease stress. Anesthesia was performed intravenously (18-gauge IV catheter in-ear vein) with Propofol 3 mg/kg and maintained with rocuronium 0.8 mg/kg along with inhaled isoflurane 2% via the automatic standard respiratory system. Vital parameters were monitored through a mechanic ventilator machine. Heartbeat was monitored with a pulse oximeter (Mindray PM-60). At the end of the protocol, animals were euthanized with a lethal dose of pentobarbital (40 mg/kg).

### 2.3. Surgical Procedure

Midline laparotomy and hepatic pedicle dissection were performed to isolate the hepatic artery. The artery was then ligated with a 3/0 braided suture for 90 min to obtain a model of warm ischemia [42]. The ligature was removed for 5 h in order to observe the reperfusion injury between the early and the beginning of the late stage [43].

### 2.4. Hyperspectral Imaging

A CMOS push-broom scanning hyperspectral camera (TIVITA, Diaspective Vision GmbH, Am Salzhaff Germany) was used to generate HS images which were performed with a camera-specific software module from the same company. The three-dimensional hypercube is composed of a spatial resolution (x,y) plus a third dimension with the relative reflectance of each pixel (z). The range of the wavelength detected is 500–1000 nm with a 5 nm interval, totaling 100 wavelengths for every pixel. The scanning method is allowed through a slit-shaped aperture motorized with an internal stepper motor [44]. The resolution of the hypercube is 640 × 480 pixels × 100 wavelengths. The acquisition is performed at ~40 cm of distance from the sample and monitored by a distance sensor Bluefruit Feather nRF52832 with Adafruit VL53LOx device (Adafruit, New York, NY, USA) orthogonal to the liver surface. The light source is composed of 6 halogen lamps of 20 W (OSRAM Halospot 70, OSRAM GmbH, Munich, Germany). The HS camera takes ~6 s to perform the acquisition of the hypercube which is transferred to a PC where it is processed creating pseudo-color images. The relative reflectance $\left(\frac{I}{I_{0}}\right)$ is converted in relative absorbance through the equation $A=-ln\left(\frac{I}{I_{0}}\right)$.

The device used in this experiment provides different algorithms (preset), that quantify the relative oxygen saturation (StO_2_%) of the microcirculation at a depth of ~1 mm, and at deeper layers with the near-infrared (NIR) spectrum (3–5 mm) [4]. Quantitative analysis of the StO_2_% and NIR index was performed intraoperatively using the TIVITA Suite software module over the whole liver surface. The methods and algorithms of the TIVITA system were explained in more detail by Holmer et al., in 2018 [4]. Briefly, StO_2_% is calculated with an algorithm based on the second derivative of the absorption spectra (570–590 nm and 740–780 nm). The NIR perfusion index is calculated with the absorbance spectra in a spectral range of 655–735 nm and 825–925 nm.

### 2.5. Artificial Intelligence-Based Analysis

#### 2.5.1. Overview

Two CNNs were created to perform an automatic HS image analysis (Figure S3). The first CNN was trained to automatically recognize liver tissue in any HS image. We refer to this as the organ segmentation CNN. The second CNN was trained to automatically characterize liver tissue which has been recognized by the organ segmentation CNN into two classes, namely perfused and ischemic liver. We refer to this as the tissue characterization CNN (Figure 1c).

#### 2.5.2. Organ Segmentation CNN and Post-Segmentation Filtering

The organ segmentation CNN operated as follows. For each spatial coordinate (x,y) in the HS image, an HS subvolume centered at (x,y) with a spatial window of 5 × 5 pixels was extracted. The subvolume had the following dimensions: 5 × 5 × 100, with two spatial dimensions and one wavelength dimension of 100 bands. The subvolume was then passed to the organ segmentation CNN, which outputted a binary classification value. Either a value of +1 (positive) or −1 (negative) was outputted, corresponding to a prediction of a liver or non-liver structure occurring at a spatial location (x,y) respectively. Predictions were made for all spatial locations, generating a spatial map (also known as a segmentation mask). Finally, post-segmentation filtering was performed to eliminate spurious regions in the segmentation masks (typically produced by specular reflections). This was achieved with a connected component analysis to identify the largest region in the segmentation mask, and all positive pixels which did not belong to this largest region were then removed. This filtration stage eliminated small positive ‘islands’ in the segmentation mask which are morphologically unlikely to be liver tissue. Secondly, any small holes that were present in the segmentation mask were automatically filled using the image morphological operations with OpenCV (https://opencv.org/, accessed on 1 March 2021). An example of a segmentation mask before and after the filtering stages is shown in Figure S2a.

#### 2.5.3. Tissue Characterization CNN

All positive spatial locations within the segmentation mask were then processed by the tissue characterization CNN. This computed a binary classification where +1 indicated ischemic and −1 indicated healthy liver respectively at each spatial location. Analogous to the organ segmentation CNN, its input is a sub-volume extracted from the HS image of 5 × 5 × 100 in size, centered at a given spatial location. Classification was performed for all spatial locations, generating a tissue characterization mask. Finally, a single ischemic score for each image was computed by taking the proportion of pixels with positive detections divided by the total number of pixels in the segmentation mask.

#### 2.5.4. CNN Design and Implementation

The two CNNs have identical architecture and they are based on a state-of-the-art 3D CNN [45]. Architecture version 6 from reference 60 was selected because it is a relatively small CNN, so it can be trained with small datasets, yet it can also learn effective multi-scale spatio-spectral segmentation features in HSI data. This was demonstrated by its good performance for segmenting remote sensing HSIs with limited data, The CNN is organized such that in early layers, 3D spatio-spectral feature maps are produced, which are then reduced to1D feature vectors, and then finally processed by a fully connected layer. Network parameters are significantly reduced using ideas from SqueezeNet (https://arxiv.org/abs/1602.07360, accessed on 1 March 2021) [46], using a small number of filters combined with 1D convolutions along the spectral dimension in the pooling phase. We illustrate the architecture in Supplementary Figure S3. Layer 1 is a 3D convolution with 20 output channels. Layer 2 is a 1D convolution along the spectral dimension with pooling using a stride of 2. Layers 3 and 4 replicate layers 1 and 2 with 35 output channels. Layers 5 and 6 are 1D convolutions with pooling to further reduce the spectral dimension. Layer 7 is a fully connected layer where the number of output neurons is the number of classes (in our case 2). Layers 1, 3, 5, and 6 are followed by ReLU activation and layer 7 is followed by sigmoid activation. The CNN has a total of 32,444 trainable parameters and it was implemented in Pytorch 1,4.0 (https://pytorch.org/, accessed on 1 March 2021) with Python 3.7.4 (https://www.python.org/, accessed on 1 March 2021).

The CNN was organized into 7 layers, where each layer consisted of a spatial convolution followed by downsampling and max-pooling operations. The penultimate layer was fully connected, and the final layer had two neurons corresponding to positive and negative classes. The CNNs were implemented in Pytorch 1.4.0 with Python 3.7.4. The CNNs have 333,560 weights which were automatically learned in the training processes.

#### 2.5.5. AI Training Processes

**Figure 2:**
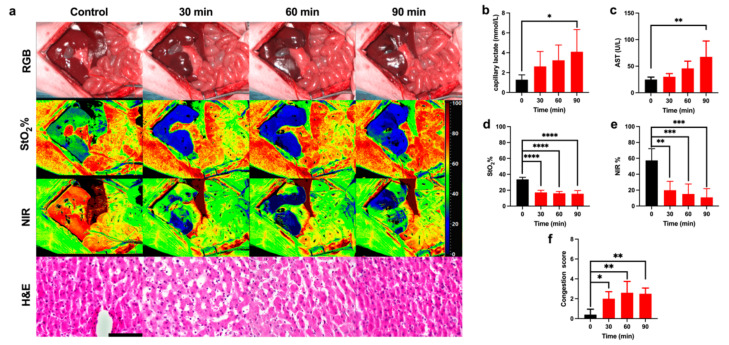
Ischemic phase evaluation. (**a**) RGB, HSI, and H&E images of the liver during the ischemic phase. Hyperspectral images of StO_2_% and NIR% showed an oxygenation decrease during the ischemic phase. H&E images show the gradual congestion increase. (**b**,**c**) Capillary lactate (*n* = 5) and AST (*n* = 4) levels indicated a gradual liver impairment. (**d**,**e**) Both preset software parameter indexes of the oxygenation showed a significant decrease in oxygen levels. (**f**) The histopathological score of congestion was significant during the ischemic phase. Data are compared to the control, ns *p* > 0.05, * *p* ≤ 0.05, ** *p* ≤ 0.01, *** *p* ≤ 0.001, **** *p* ≤ 0.0001 (*n* = 5). Histology photos were taken with a Leica DM2000 LED microscope, magnification 40×, scale bar 100 μm.

The organ segmentation CNN was trained from segmentation masks generated from HS images by a skilled human operator. The operator demarcated the spatial extent of the liver in each HS image, for all pigs and at times 0 min, 30 min, 60 min, and 90 min (42 images). An interactive segmentation software was used (GIMP v.2.8), using an RGB image simulated from each HSI image. As an example, we illustrated a demarcated image (Figure 2b,f). The operator segmented both the interior of the liver (positive class) and all regions surrounding the liver (negative class). The organ segmentation CNN was then trained using supervised learning with the segmentation masks provided by the human operator as ground truth. Special care was taken to ensure that the ability of the organ segmentation CNN to generalize to novel subjects could be tested. To achieve this, LOPOCV was performed as follows. Five organ segmentation CNNs were trained, with one CNN for each pig. Each CNN was trained using image data from all pigs except for one (the held-out pig). The predictive performance of the CNN was then evaluated using images from the held-out pig only. LOPOCV was necessary to eliminate an elevated performance bias, which occurs if a CNN is trained and evaluated on data from the same subject.

The training was performed and implemented in Pytorch using as follows. The HSI images used for training were first concatenated vertically to form a single HSI image, denoted by the HSI image *I*. The training label images were similarly concatenated to form a single training label image *L*. The values in *L* were either +1 (indicating liver), −1 (indicating non-liver), or 0 (indicating a spatial location that was close so close to the border of the liver that its label could not be determined by the annotator thanks to optical blurring around the liver border. Only pixels labeled +1 or −1 were used for training. Class-balanced binary cross-entropy was used as the loss function, implemented by inverse median weighting. Specifically, the loss of each class $i\in \left[-1,+1\right]$ was weighted by the value $\frac{m}{f_{i}}$ where $f_{i}$ denotes the proportion of pixels labeled as class i in L and $m=\frac{1}{2}\left(f_{-1}+f_{+1}\right)$. The CNN weights were initialized with Kaiming initialization [47] and biases were initialized to zero. At each training epoch, a batch of 5 × 5 × 100 subvolumes were randomly selected without replacement from *I*. Each subvolume was positioned in *I* such that its center was at a random spatial location in *I* and whose label in *L* was either −1 or +1. A batch size of 8192 was used. The CNN parameters were updated from each batch with stochastic gradient descent (SGD) using the implementation from Pytorch’s “torch.optim” package with a learning rate of 0.01 and a weight decay (L2 regularization) of 0.0005. The training was terminated after 2000 epochs. The time to train was approximately 13 h using a server (DGX 1 equivalent, Nvidia cooperation). The time to train all 5 CNNs for each cross-validation was approximately 65 h.

The tissue characterization CNN was trained in practically the same manner as the organ segmentation CNN. Specifically, all training parameters and processes were identical, and the sole difference was the construction of the training data. LOPOCV was also used where for each animal, one tissue characterization CNN was trained using HSI data from all other animals (4 images per animal corresponding to times 0 min, 30 min, 60 min, and 90 min). Its predictive performance was then evaluated on the 4 images from the held-out animal. The histology score (congestion score) together with the surgical procedure was used as the ground truth. The HSI images used for training were concatenated vertically to form a single HSI image *I′* with a corresponding label image *L′.* A value of *L′*(x,y) = +1 was used if the pixel at spatial location (x,y) was annotated as liver and the liver was determined as ischemic from the histology score. A value of *L′*(x,y) = −1 corresponded with an annotated liver pixel that was determined to be healthy from the histology score. A value of *L′*(x,y) = 0 corresponded to all other pixels, and those pixels were not used during training. The training was then performed exactly as described for training the organ segmentation CNN.

### 2.6. Blood Analysis

Blood for systemic lactate and liver function was sampled through a central catheter placed in the jugular vein (6 French IV catheter). Capillary lactate was analyzed by puncturing Glisson’s capsule with a randomized selection of liver segments. Capillary and systemic lactates were measured using a strip-based portable lactate analyzer, which presents a margin error of 0.35 mmol/L (EDGE, ApexBio, Taipei, Taiwan). The correlation analysis of the data was performed between HSI parameters and capillary lactate concentration. The surgical intervention was monitored to rule out any bias in the hepatic ischemic phase by means of systemic blood gas analysis (BGA) with the epoc Blood Analysis System (Siemens Healthineers, Henkestr, Germany) to measure pO_2_, pCO_2_, pH, glucose, creatinine, urea, and BUN. Liver functionality was assessed by means of aspartate aminotransferase (AST), alanine aminotransferase (ALT), prothrombin time (PT), gamma glutamyl-transferase (GGT), alkaline phosphatase (ALP), total protein (TP), and albumin analysis. Liver injury after the reperfusion phase was assessed also via AST and ALT.

### 2.7. Histology

Liver biopsies were randomly taken with a 16 G biopsy gun (Temno Biopsy System, Galway, Ireland) from the posterior segments at each timepoint. Sections of 5 μm were taken from formalin-fixed paraffin-embedded blocks and were dewaxed and rehydrated prior to staining at room temperature. A treatment with hematoxylin Harris’ formula (Leica Biosystems, Muttenz, Switzerland) for 10 min and then a wash in acid alcohol for 2 s and in tap water for 2 min were performed. Eosin staining with Eosin 0.5% (Leica Biosystems, Muttenz, Switzerland) for 3 min was performed before washing in tap water for 30 s. Finally, the sections were dehydrated with ethanol 100% and placed in xylene until their mounting with coverslips. A semi-quantitative blinded analysis was performed by a pathologist using Suzuki’s criteria [48].

### 2.8. Immunohistochemistry Staining

Sections were fixed in 10% neutral buffered formalin (NBF) and processed for histological examination, which included paraffin embedding, sectioning, and staining with hematoxylin and eosin. Sections from selected paraffin blocks for each specimen were used for immunohistochemical analysis. Slides of 4 μm-thick tissue sections were incubated at room temperature in an antigen retrieval process (EDTA citrate buffer, pH 8.3, CC1 buffer), revealed with ‘Ultra View’ Universal DAB Detection Kit and counterstained with a hematoxylin solution (Ventana Roche Systems, München, Germany). They were treated on automated VENTANA-Benchmark-XT with CD31 (rabbit monoclonal, EP78 clone, Microm; pre-treatment: CC1 36 min; dilution: 1/200 during 32 min).

### 2.9. Confocal Endomicroscopy

Confocal endomicroscopy was performed on the anterior surface (the same analyzed via HSI) of Glisson’s capsule at the end of the procedure. This test evaluated blood microcirculation and arterial supply in randomly selected segments with Cellvizio pCLE system (Mauna Kea Technologies, Paris, France). Confocal images were obtained by intravenously injecting 2 mL of sodium fluorescein (Fluocyne, Serb, Paris, France).

### 2.10. Statistical Analysis

Statistics were performed using GraphPad 8.3 (GraphPad Software, San Diego, CA, USA). A Spearman’s and Person’s rho were analyzed to perform the correlation between optical and biological data. All data were expressed as means ± s.d. One-way ANOVA with Dunnett’s multiple comparisons was performed for parametric tests to calculate differences in continuous paired variables. The Friedman test with Dunn’s multiple comparison test was applied for paired non-parametric tests. A two-tailed *p*-value < 0.05 was considered statistically significant. One-tailed significant *p*-value < 0.05 was applied to the confusion matrix in the correlation between the AI score and biological data, considering that the correlation among these values was physiologically possible only in one direction.

## 3. Results

### 3.1. Ischemic Phase

The surgical intervention was performed under general anesthesia with continuous monitoring of vital parameters (Figure S1). No significant impairments of vital parameters were found during the ischemic phase. However, a significant change in urea and blood urea nitrogen (BUN) was found, although the creatinine level showed no significant changes (Figure S1a–o). HSI images showed an oxygenation decrease in both indexes (StO_2_% and NIR%) during the ischemic phase, in congruence with the histological assessment (Figure 2a). Before ligation, the perivenular region presented a regular hepatocellular muralium without any ischemic morphological alteration. After 90 min of ischemia, the parenchyma was characterized by weaker staining in some of the hepatocytes’ cytoplasm, which appeared pale with a reduced volume and hyperchromatic nuclei. Capillary lactate and AST showed a significant increase after 90 min of ischemia (4.1 ± 2.23 mmol/L, *p* = 0.0102 and 67.50 ± 29.89 U/L, *p* = 0.0061 respectively) (Figure 2b,c). The quantitative analysis of StO_2_% and NIR% showed a significant decrease with a minimum at 90 min (15.57 ± 3.98%, *p* < 0.0001 and 10.90 ± 11.00%, *p* < 0.001 respectively) (Figure 2d,e). The ischemic phase was confirmed by the congestion score, which was significantly higher with a plateau phase and a maximum at 60 min (2.5 ± 0.577 a.u., *p* = 0.0015) (Figure 2f).

**Figure 3:**
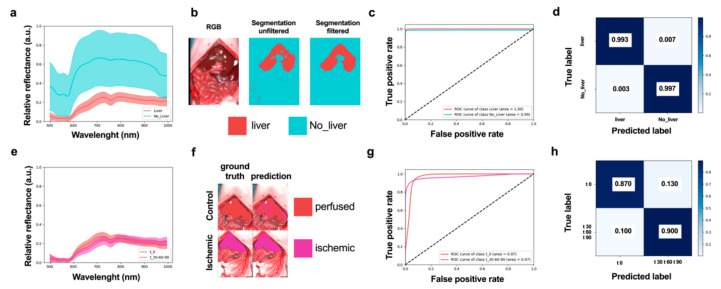
AI-based training and evaluation in the ischemic phase. (**a**) Liver and non-liver spectral distributions showing mean (central curve) and 1 s.d. (region). (**b**) Visualization of automatic tissue segmentation of liver and non-liver classes with and without post-segmentation filtration. (**c**) Receiver operator characteristic (ROC) curves corresponding to liver and non-liver classes, showing the trend relationship between false positive and true positive rates. (**d**) Confusion matrix showing specificity and sensitivity of tissue segmentation CNN. (**e**) Spectral distributions of perfused and ischemic classes. (**f**) Visualization of tissue automatic tissue characterization results with two classes (perfused and ischemic). (**g**) ROC curves of tissue characterization CNN showing the relationship between false positive and true positive rates. (**h**) Confusion matrix showing specificity and sensitivity of tissue characterization. (*n* = 5).

A convolutional neural network (CNN) was trained to automatically classify each pixel of HS images into two classes: liver tissue and non-liver tissue/other structures (called liver segmentation CNN) (Figure 3a–c and Figure S2a). The performance of liver segmentation CNN was measured with HSI images from the control and ischemic phases using leave-one patient-out cross-validation (LOPOCV). Class predictions (labels) were made for each pixel in each HS image, and predictions from all images and all pixels were combined to produce a normalized confusion matrix (Figure 3d). A sensitivity of 0.993 and a specificity of 0.997 were achieved (top left and bottom right entries in the confusion matrix respectively), showing that the CNN could very accurately discriminate liver tissue from other organs in all images.

All pixels which were classified as liver by the liver segmentation CNN were processed by the tissue characterization CNN to automatically predict if the liver was perfused or ischemic (Figure 3e–g). Predictions were accumulated for all pixels and all HS images, and performance was evaluated with a normalized confusion matrix (Figure 3h). This shows a strong potential of the tissue characterization CNN to discriminate between perfused and ischemic liver during the surgical procedure with high sensitivity (0.870) and specificity (0.900). CNN predictions (AI score) were coherent with the experimental workflow. Indeed, the ischemic phase was significantly lower after 30 min of occlusion when compared to the control, and this difference was maintained after 60 and 90 min (*p* < 0.0001 for all timepoints) (Figure S2b,c).

### 3.2. Reperfusion Phase

**Figure 4:**
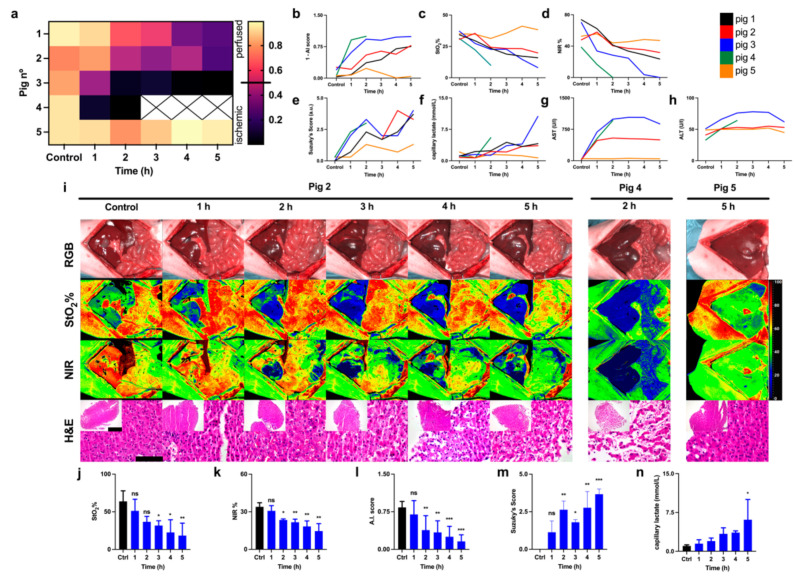
Reperfusion phase. (**a**) Automatic AI score of liver viability for each pig for control and reperfusion times. (**b**) Visualization of AI scores for each pig as a function of time (*y*-axis was flipped to provide a better visual comparison with Suzuki’s score (**c**,**d**) StO_2_% and NIR indexes (*n* = 5). (**e**) Suzuki’s score (*n* = 5). (**f**) Capillary lactate (*n* = 5). (**g**) AST (*n* = 4). (**h**) ALT (*n* = 4). (**i**) HSI images and H&E images. Pig 2 showed a gradual parenchymal disruption; pig 4 died after 2 h, HSI and H&E showed tissue ischemia and parenchymal disruption respectively. Pig 5 was healthy at the end of the procedure; here, HSI showed a perfused liver and H&E confirmed normal parenchyma. Pigs 1, 2, 3 were grouped (*n* = 3). (**j**,**k**) Indexes of StO_2_% and NIR showed a significant decrease together with (**l**) the AI score. (**m**) Suzuki’s score and (**n**) capillary lactates showed an opposite significant trend. Data are compared to the control, ns *p* > 0.05, * *p* ≤ 0.05, ** *p* ≤ 0.01, *** *p* ≤ 0.001, (*n* = 5). Histology photos were taken with a Leica DM2000 LED microscope, magnification 40× and 10×, scale bars 100 μm and 200 μm respectively.

CNNs were evaluated on each reperfusion HS image using LOPOCV. The workflow was identical to the processing of the HS images in the control and ischemic phases. First, the liver recognition CNNs detected pixels belonging to the liver, and these were then processed by the tissue characterization CNN to assess perfused or ischemic liver pixels. For each HS image in control and reperfusion phases, a global perfusion AI score was computed as the proportion of detected perfused pixels as compared to the total number of detected liver pixels. A prediction score of 0 indicated total ischemia and 1 indicated total perfusion. The scores for all pigs and timepoints are visualized in Figure 4a,b. The prediction score showed a common outcome for pigs 1, 2, and 3 (Figure 4a). The score decreased gradually with a minimum value at 5 h of reperfusion (0.246, 0.222, 0.009 a.u. respectively for pig 1,2,3) (Figure 4b). Pig 4 died after 2 h of reperfusion with a score of 0.003; pig 5 had the highest score of 0.959 at the end of the procedure, similar to the control (0.940). Pigs 3 and 5 showed an opposite outcome after 5 h of reperfusion. StO_2_% and NIR% parameters were lower in pig 3 and higher in pig 5 (8.14 ± 0.97, 38.32 ± 6.59 StO_2_% and 0.04 ± 0.86, 47.20 ± 6.90 NIR%) (Figure 4c,d). Suzuki’s score presented a maximum in pig 3 and a minimum in pig 5 (4.0 and 1.3 a.u. respectively) similarly to the AI score (Figure 4e).

Save from pig 5, all pigs showed a gradual damage increase. Capillary lactate was higher in pig 3 and lower in pig 5 (10.60 and 0.70 mmol/L respectively) (Figure 4f). Similarly, AST and ALT were high in pig 3 (879.00 U/L, AST, 62.00 U/L, ALT) and low in pig 5 (46.00 U/L, AST, 45.00 U/L, ALT) (Figure 4g,h). In RGB images, which closely correspond to the human eye evaluation, global color changes were slightly visible after 4 h of reperfusion (Figure 4i). The imaging of hyperspectral parameters (StO_2_%, NIR%) appeared similar at time 0 (before ligation) and after 1 h of reperfusion. Considering that the apparent red color corresponds to the maximum perfusion and the blue color to the minimum one, after the second hour of reperfusion, all parameters gradually decreased, reaching a minimum after 5 h similarly to the level showed after 90 min of ischemia (Figure 4a). StO_2_% presented a higher level of ischemia as compared to NIR% (more diffused and darker blue). Setting NIR% against StO_2_% images, NIR% presented sharper limits of the ischemic area, and a higher signal after the first hour of reperfusion was found. The HS data showed more visible and well-defined changes as compared to the RGB camera. Oxygenation measured with StO_2_% and NIR% significantly decreased in both ischemic and reperfusion phases. The last HS image of pig 4 that died after 2 h of reperfusion showed that the liver was not perfused, and H&E confirmed parenchymal and microvascular disruption. Overall, after 5 h of reperfusion, the perivenular region appeared necrotic, probably due to the ischemic damage with a detachment of hepatocytes from the adjacent sinusoidal reticular support. Hepatocytes were reduced in volume with acidophilic cytoplasm and pyknotic nuclei. A mix of inflammatory cells such as lymphocytes and polymorphonuclear elements were visible in the parenchyma (Figure 4i). Grouping pigs 1, 2, and 3, following the AI score, the statistical analysis showed that the HS image quantification of StO_2_% and NIR% presented a significant decrease as compared to the control (*p* = 0.0014 StO_2_%, *p* = 0.0003 NIR%) (Figure 4j,k). The AI score showed a gradual decrease with a minimum after 5 h (0.160 ± 0.130 a.u., *p* < 0.0001) where Suzuki’s score and capillary lactate concentration were significantly high (3.667 ± 0.3512 mmol/L, *p* < 0.0001 and 6.1 ± 3.9 mmol/L, *p* = 0.0228 respectively) (Figure 4l–n).

**Figure 5:**
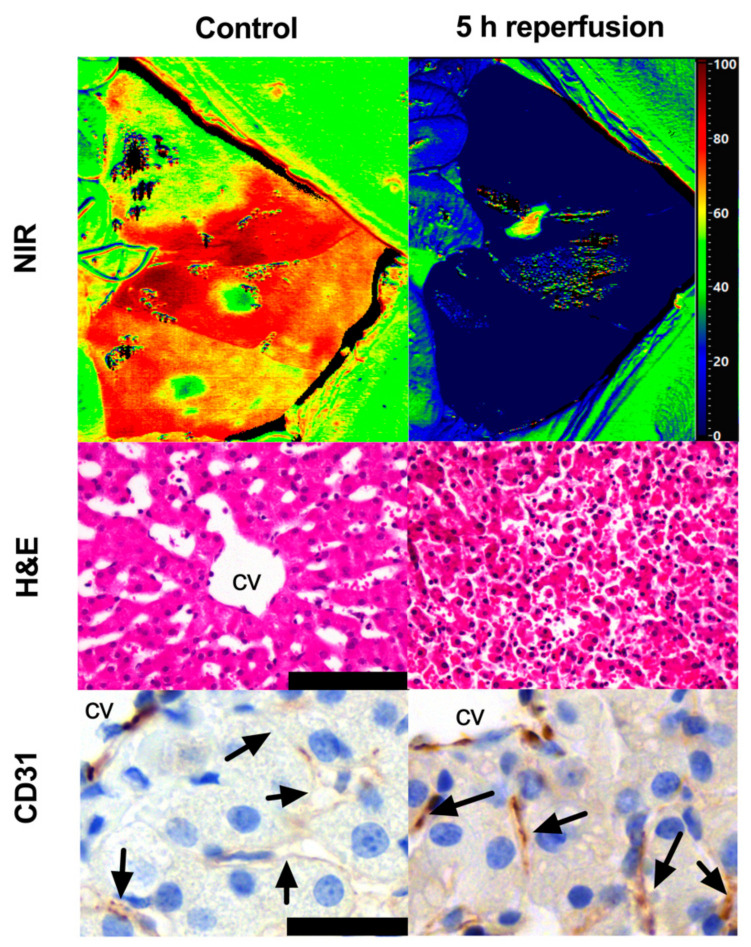
HSI, H&E, and IHC of CD31 expression comparison. The staining for CD31 did not show sinusoidal staining in the control group; this group corresponded to a high level of oxygenation in the HSI image and a normal microarchitecture of the lobe in H&E. After 5 h of reperfusion, a higher expression of CD31 was observed close to the central vein, which corresponded to a low level of oxygenation in the HS image, a deeply congested microvasculature, and an overall parenchymal disruption in H&E. Histology photos were taken with a Leica DM2000 LED microscope, magnification 40×, scale bar 100 μm (H&E), 160×, scale bar 25 μm (CD31).

Immunostaining for CD31 revealed stronger staining after 5 h of reperfusion as compared to the control. HSI, H&E, and IHC were displayed together for better visualization of the difference between the control and the damaged liver from different perspectives (Figure 5). Pig 4 showed strong CD31 staining at 2 h and in the control (Figure S2d). Pig 5 showed a similar expression in the control and after the reperfusion phase (Figure S2e). Endomicroscopy confirmed the presence of blood circulation at the end of the reperfusion phase as shown in pigs 3 and 5 (Video S1,2 respectively for pigs 3,5). The liver parenchyma appeared perfused in both pigs. However, pig 3 presented areas characterized by a slower to absent blood flow on which the microcirculation system appears compromised. Both pigs showed a correct vascular flow in the larger arterial and portal branches of the randomly selected lobes.

### 3.3. Correlation of HSI with Capillary Lactate

**Figure 6:**
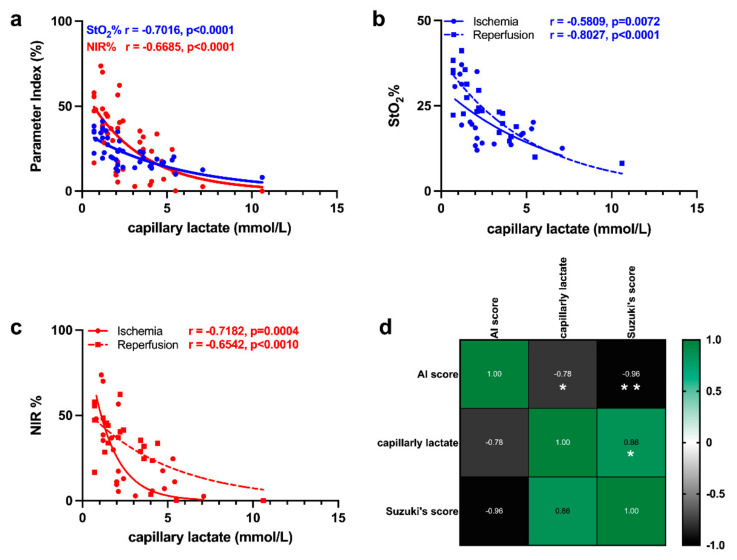
Correlation between optical and biological data. (**a**) Correlation between StO_2_%, NIR, and capillary lactates. (*n* = 42) (**b**,**c**) StO_2_% and NIR correlation split into ischemic and reperfusion phases (*n* = 42). (**d**) Correlation matrix of AI score, capillary lactates (mmol/L), and Suzuki’s score (*n* = 22 per each variable for 5 pigs). Data are expressed as mean ± s.d. Spearman’s and Pearson’s correlation significance: * *p* ≤ 0.05, ** *p* ≤ 0.01.

The correlation of HSI indexes with capillary lactate was significant and higher for StO_2_% as compared to NIR% (Figure 6a). When the images were split into ischemic and reperfusion phases, StO_2_% and NIR% showed different degrees of correlation. StO_2_% correlated better with the reperfusion phase and NIR% showed a higher correlation for the ischemic phase (Figure 6b,c). Finally, the AI score of the reperfusion phase was negatively correlated with capillary lactates and Suzuki’s score (r = −0.78, *p* = 0.0320 and r = −0.96, *p* = 0.0012). Additionally, Suzuki’s score and capillary lactates were positively correlated (r = 0.86, *p* = 0.0135) (Figure 6d).

## 4. Discussion

### 4.1. Ischemic Phase

In this study, we explored the potential of the AI-HSI analysis to predict liver viability following a period of 5 h of reperfusion phase after 90 min of hypoxia performed through the occlusion of the hepatic artery [49]. The warm ischemic period was found to be enough to obtain parenchymal damage worsened by the reperfusion phase [42]. Hemodynamic parameters, pH, and systemic oxygenation remained stable during ischemia. A small increase in pCO_2_ was found. However, it was not statistically significant probably due to its continuous delivery into the systemic circulation (Figure S1a–d) [28]. The glucose level was constant during the ischemic phase, in congruence with the literature (Figure S1e) [27]. Liver ischemia-reperfusion injury is known to be the leading cause of acute kidney failure [50]. Although the creatinine increase was not significant, the BUN and urea increase was statistically significant, suggestive of an early kidney dysfunction according to a previous study (Figure S1f–h) [51]. No significant changes were found in liver functionality and systemic lactate levels (Figure S1i–o).

HS images could identify the ischemic areas showing the potentiality to exceed the capabilities of human vision which can distinguish only three main ranges corresponding to cone visual pigments (from ~424 to ~563 nm) [52,53]. In our study, HS image AI analysis used 100 bands from 500 to 1000 nm. The following interpretation and translation of relative reflectance quantification at different bands into RGB visible changes via the algorithms enhance the ability of the human eye to evaluate physiological changes (Figure 4i). The image capture took only ~6 s to be ready for its interpretation, which is significantly much faster than any other type of clinically available intraoperative assessment tool. In addition, its simple application reduces the variable of the operator’s experience to a marginal value, providing a fast and standardized data extraction. Processing time for each HS image was approximately 1 s, indicating a minimal and acceptable delay to the surgeon for a potential intraoperative tool.

The overall analysis of the ischemic phase confirmed the HAO model (Figure 2 and Figure S1). StO_2_% and NIR% indexes showed that ischemia was visible during HAO as compared to the control (Figure 2a). StO_2_% showed a larger ischemic area, probably due to the higher arterial blood flow which characterizes the hepatic tissue immediately beneath Glisson’s capsule, which corresponds to the depth of StO_2_% analysis (~1 mm) [54,55]. NIR% images showed sharper limits of the ischemic area, probably because at a depth of 3 to 5 mm, the lobe is characterized by an increased arterial and portal branch, which almost equally contributes to the oxygen supply [4]. This dual-depth analysis allows HSI to show the differences in oxygenation and may help for a more comprehensive evaluation of the hepatic microvascular circulation. The gross estimation of the mean reduction of StO_2_% and NIR% was ~50%, which is close to the theoretical hepatic artery oxygen supply to the liver [56]. The plateau phase highlighted by the congestion score was probably due to the small flow maintained by the portal tract into the inferior vena cava (IVC) (Figure 2f).

Although the occlusion of the hepatic artery in the pig 5 was confirmed, as shown by the observation made by the surgeon to check any anatomical variance, by the increase in AST and local lactate (from 26.00 to 43.00 U/I, from 2.10 to 4.10 mmol/L respectively), the AI-score assigned a high value like the control. This anomaly was confirmed by histopathological analysis, CD31, confocal endomicroscopy, and blood tests which showed the absence of microvascular failure. The reason why Pig 5 had a positive response to the ischemic insult is not clear, but it was an interesting case that showed the ability of the AI-score to predict a good outcome that could be considered as a positive control.

### 4.2. Reperfusion Phase

Five hours of reperfusion phase were considered sufficient for the observation of possible hepatic damage (IRI early phase) and more than the intraoperative time necessary for hepatic surgery [43]. Except for pig 5, systemic lactate levels showed an overall increase, probably due to the flushing out of the presinusoidal capillary lactate into the IVC after the re-establishment of arterial circulation (Figure S2f). Capillary lactate levels supported the findings of hypoxia and the occurrence of the anaerobic metabolism during the ischemia and reperfusion phases. The rapid decrease after the first hour of reperfusion was probably due to the washout of the arterial blood flow. AST and ALT values were higher after the first hour of reperfusion due to the inflammatory response and the hepatic vascular damage observed in HSI images. The acute experimental design may have contributed to the lack of a massive damage spread, which is likely to continue to develop during a longer observation period (late IRI).

Oxygenation in both indexes appeared similar before ligation and after the first hour of reperfusion, showing no statistically significant difference. This confirmed the re-establishment of arterial blood perfusion. After 2 h of reperfusion, a visible decrease of oxygenation was detected, indicating the beginning of vascular dysfunction. The gradual reduction in oxygenation reached the same level of the ischemic phase at the end of reperfusion in both oxygen indexes (Figure 4i). This indicates that, after 5 h of reperfusion, the vascular system was heavily compromised. The portion of the small intestine analyzed in HSI images did not show any congestion, confirming that the portal branch was not occluded [57].

When the sinusoids are damaged, the capillarization effect occurs highlighted by the expression of CD31 [58]. Capillarization impedes the normal perfusion of molecules of blood circulation from the lumen of sinusoids. The pericentral zone of the lobe is usually the first one to be positive to CD31 given its distance from the portal triad, which increases the sensitivity to low oxygenation [59,60]. In this study, LSECs appeared to express a higher level of CD31 after 5 h of reperfusion when compared to the control (Figure 5). Pigs 5 and 4 showed low to absent and high levels of CD31 staining in the control and after the reperfusion phase respectively. This highlights the absence of capillarization in pig 5 and a capillarization effect in pig 4. The capillarization of pig 4, confirmed by the necrosis and microvascular disruption found in H&E in the control may contextualize the early liver failure (Figure S2d,e). These results confirm the AI viability score and corroborate the fact that when the AI-HSI score assigned a low score in the reperfusion phase, this was associated with microvasculature damage. Future studies could be based on the histopathological analysis damage of the whole organ, and this would help for the quantification of the amount of necrotic parenchyma over the healthy tissue. In our study, small biopsies were taken to prevent any damage to large vascular branches which could introduce a bias in the HSI analysis.

Bile presents a spectra profile, which may interfere with the preset parameters of the HSI camera which, to the best of our knowledge, are not compensated in order to obtain absolute values of oxygenation close to the real ones [31]. Nevertheless, the correlation of NIR% and StO_2_% with capillary lactates, which resulted in a significantly negative correlation confirming the adequacy of the spectra sampling (Figure 6a–c). Consequently, it may be possible to measure these two indexes as major indicators and predictors of capillary lactates even though their absolute values may be influenced by bile flow fluctuation. We also consider that the “bile effect” is probably of limited relevance in our study, considering that the bile duct was left open. The AI score, which analyses the relative reflectance of the full spectra (500–1000 nm) and is not based on proprietary preset, showed a significant negative correlation with capillary lactates and Suzuki’s score (Figure 6d). This correlation suggests that the analysis of the optical properties of the liver can infer organ viability in this scenario. The possibility to discriminate between the ischemic phase and the reperfusion damage would be the next appropriate step.

Dataset size is a very important factor when applying a deep learning approach. This relatively small dataset prohibits end-to-end training of large CNNs with a large number of trainable parameters. However, the test performances give strong empirical evidence that a deep learning approach works well with this dataset despite its small size using the proposed CNNs. We emphasize that using LOPOCV, data from the held-out animal was never used for training. The strong results, particularly liver classification with a sensitivity of 0.993 and a specificity of 0.997, show that the CNNs have learned well because they generalize to data from animals not present in the training set. This has been possible because the CNNs have been trained with mild regularization (weight decay), but more importantly, the CNNs have a relatively small number of trainable parameters (32,444). This greatly reduces the potential to overfit and it is much smaller compared to larger CNNs typically used for image classification with RGB images such as ResNet50 [61] with over 23 million trainable parameters. Recall that with RGB data, spatial context is extremely important for good classification, which requires filters acting over relatively large spatial distances. In contrast, in HSI, the rich reflectance information reduces the need for spatial context. As a future work, we aim to study the benefit of enlarging the capacity of the CNNs with a larger dataset. This can be both in terms of spatial window (to add more spatial context information) and in terms of network depth (to add more abstract spatio-spectral feature representations into the CNNs). Concerning the CNN model, we believe that a simpler classifier could also be used to perform well on the segmentation task. However, our work shows that the exact same machine learning model (CNN) can be used to effectively solve both tasks without any modification to the model’s design or training process, which adds simplicity with a unified approach. As a follow-up study, we aim to compare performance from the CNNs with several other machine learning models such as Support Vector Machines (SVMs) or logistic regression, to investigate how performance is affected by the specific choice of machine learning model.

In clinical routine, HAO is evaluated with US and CT in combination with blood analysis. US is still the best clinical tool for postoperative HAO detection. A recent comparison found that US was more accurate with a significantly higher specificity [62,63]. Despite its advantages, for patients with no visible hepatic artery flow, angiography or a contrasted CT scan is performed to obtain a clear diagnosis. Overall, these techniques present their own drawbacks and limitations. CT scan has contraindications such as nephrotoxicity because of iodine contrast medium injection and allergic reactions. When arterial revascularization is performed, there is a “grey zone” where it is not possible to predict the future graft function and the potential consequences of biliary ischemia. In contrast, US is operator-dependent and requires intensive training to be mastered. The evaluation may be difficult due to a poor penetrance in case of distended bowel loops or obesity, and due to anatomical variations. US cannot provide a precise global map of liver oxygenation intraoperatively.

The need for an objective and convenient HAO analysis tool has driven researchers to find alternative solutions. Pischke et al. reported a useful methodology for HAO assessment by using an IscAlert PCO_2_ sensor for carbon dioxide [28]. They were able to provide a real-time monitoring system, which could discriminate arterial from portal occlusion. Although this approach was accurate and promising, it required the insertion of a catheter, making this application slightly invasive. Additionally, the analysis of the level of carbon dioxide can provide the level and type of hypoxia only, but not its localization.

The development of imaging methods such as fluorescence-based perfusion assessment via the injection of indocyanine green (ICG) and near-infrared spectroscopy (NIRS) showed promising results [23,24,25,26]. ICG clearance is achieved by the hepatocytes in the parenchyma and excreted mostly in the bile through the enterohepatic circulation. For that reason, its application in HAO assessment is theoretically more than appropriate. Additionally, ICG was already successfully applied in the study of bowel perfusion [64]. In 2011, Levesque et al., in a study on 14 patients, showed that the ICG disappearance rate (PDR-ICG) was significantly lower in patients with early and late HAO than in patients without HAO in a HAT context [23]. Although the ICG PDR-based assessment showed positive results, it has drawbacks such as the need for an injection, the time required for clearance, the contraindications in iodine allergic patients, or those affected by thyrotoxicosis, and its application in the clinical setting is not allowed in many countries. The application of NIRS was the first approach for a non-invasive analysis of hemoglobin without the need for the injection of an exogenous compound for the early diagnosis of HAO. In 2016, Skowno et al. studied transcutaneous hepatic StO_2_% monitoring with the NIRS system and they reported that it was not a reliable method for the detection of hepatic ischemia. However, it was potentially useful in small pigs. Liver micro-dialysis was also tested with positive results although this technique is still invasive and can change the surgical workflow, which is not always possible [51]. Our AI HSI-based evaluation may help to predict the extent of ischemia-reperfusion injury, indicating that arterial revascularization is not sufficient to prevent liver failure or massive damage. This study could be the basis for a future prediction model of patient outcomes in a clinical context. Although the HSI system showed the ability to correlate optical and biological properties intraoperatively that ultimately made possible the prediction of liver viability, it presents several limitations.

Currently, HSI can be employed exclusively as an intraoperative tool. One possible application would be the detection of perfusion deficits after completion of the vascular anastomoses during liver transplantation. Additionally, spectroscopic probes, acting within the same range of wavelengths and with a similar algorithm, could be inserted percutaneously. US could be applied to guide the probes onto the liver’s surface providing reliable perfusion information in selected clinical cases. These cases could be the ones that are not suitable for US (poorly visible artery) or CT scan (iodine allergy or kidney failure).

The oxygenation map and the optical analysis represent only the first 3–5 mm of the tissue; hence it is not possible to detect a specific problem of the vasculature in-depth directly. Additionally, HSI cannot perform video. Our team partially overcame this problem by developing hyperspectral enhanced reality (HYPER) to guide the demarcation line assessment during the hepatectomy [29,38,39]. Finally, its ability to discriminate between ischemia and congestion is not clear as well as the ability to distinguish different ischemic timepoints. Although with its limitations, we consider that HSI-AI is a non-invasive tool that can furnish additional information on liver viability intraoperatively offering an automatic and standardized system.

## 5. Conclusions

The artificial-intelligence and hyperspectral imaging-based score could predict liver viability during the ischemic and reperfusion phases with a significant correlation with histopathological analysis, capillary lactate concentration, and CD31 staining. Further analysis with different types of vascular occlusion and with an increased number of pigs is necessary to confirm this data. HSI is a valuable non-invasive tool that can predict biological properties of the reperfusion damage produced by HAO intraoperatively.

## Data Availability

The data presented in this study are available on request from the corresponding author.

## Supplementary Materials

The following are available online at https://www.mdpi.com/article/10.3390/diagnostics11091527/s1, Figure S1: Ischemic phase monitoring. Figure S2: Additional information. Figure S3: CNN architecture. Video S1: endomicroscopy of pig 3. Video S2: endomicroscopy of pig 5.
