# Supplementary material for: Automatic Liver Viability Scoring with Deep Learning and Hyperspectral Imaging
Source: Diagnostics (Basel). 2021 Aug 24;11(9):1527. doi: 10.3390/diagnostics11091527 (PMC8472457; doi:10.3390/diagnostics11091527)
Supplement: Supplementary file 1 [file diagnostics-11-01527-s001.zip › Supplementary materials.pdf]

Supplementary Materials

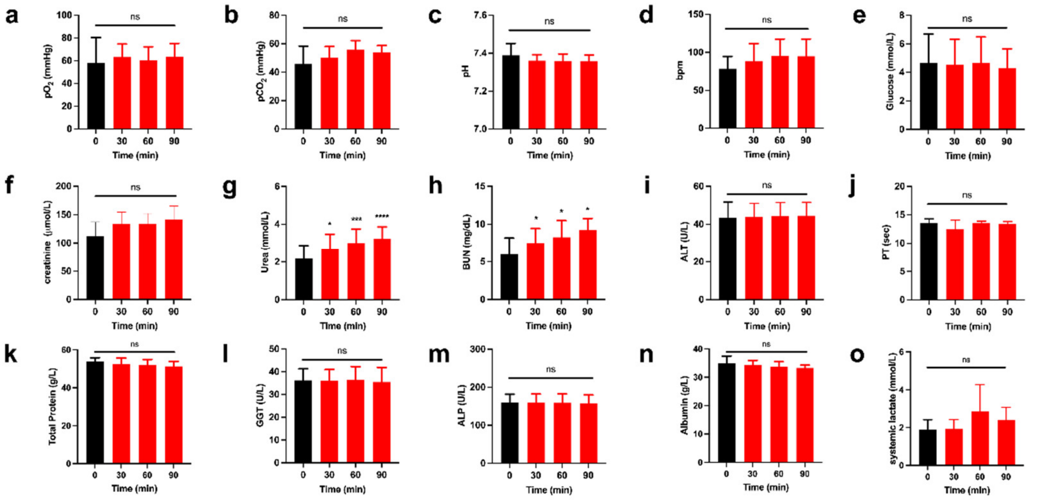

Figure S1. Ischemic phase monitoring. Blood analysis was performed to monitor the ischemic phase and the surgical maneuver. No significant alterations in blood parameters were found except for urea and BUN, which confirmed the hepatic ischemic phase (**a**) Partial oxygen pressure (n = 4). (**b**) Partial carbon dioxide pressure (n = 4). (**c**) pH Monitoring (n = 4). (**d**) Heartbeat per minute (n = 4). (**e**) Glucose (n = 4). (**f**) Creatinine (n = 4). (**g**) Urea (n = 4). (**h**) BUN (n = 4). (**i**) ALT (n = 4). (**j**) Prothrombin time (n = 4). (**k**) Total protein (n = 4). (**l**) GGT (n = 4). (**m**) ALP (n = 4). (**n**) Albumin (n = 4). (**o**) Systemic lactate (n = 5). Data are compared to the control, ns $p > 0.05$, * $p \leq 0.05$, ** $p \leq 0.01$, *** $p \leq 0.001$, **** $p \leq 0.0001$. (n = 5 pigs).

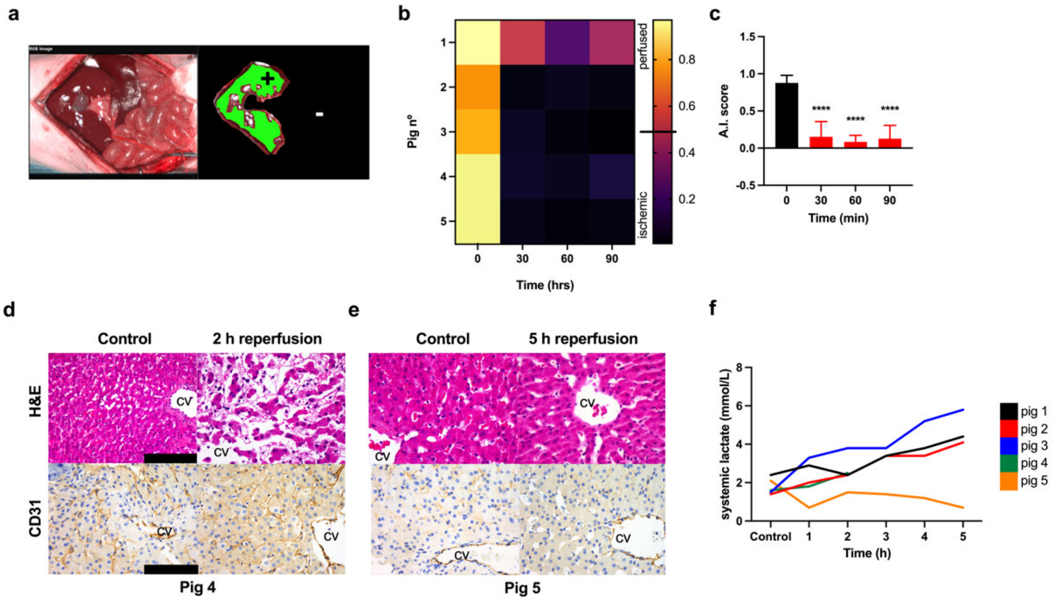

Figure S2. Additional information. (**a**) Tissue segmentation ground truth, the tissue was labelled by a skilled operator. (**b**) AI score of ischemic phase. (**c**) AI score statistics. (**d**,**e**) H&E and CD31 staining of pigs 4 and 5 before ligation and after the reperfusion phase. (**f**) Systemic lactate of the reperfusion (n = 5). Data are compared to the control, **** $p \leq 0.0001$. Histology photos were taken with a Leica DM2000 LED microscope, magnification 40×, scale bar 100 µm.

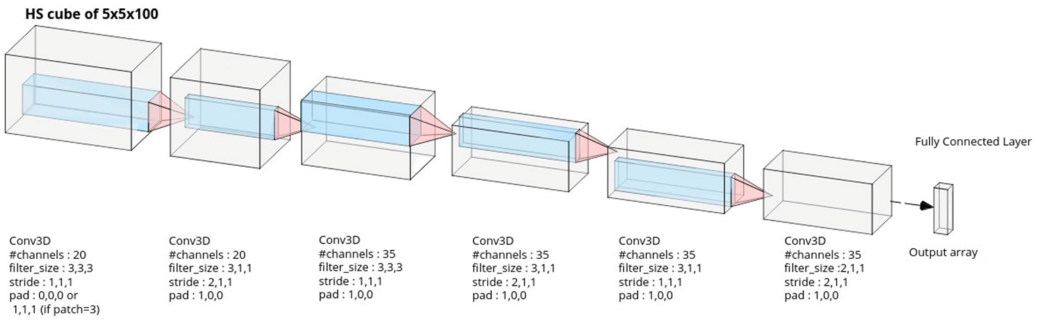

Figure S3. CNN architecture**.**
